# Dairy Intake Modifies the Level of the Bile Acid Precursor and Its Correlation with Serum Proteins Associated with Cholesterol Clearance in Subjects with Hyperinsulinemia

**DOI:** 10.3390/nu15224707

**Published:** 2023-11-07

**Authors:** Atena Mahdavi, Jocelyn Trottier, Olivier Barbier, Michel Lebel, Iwona Rudkowska

**Affiliations:** 1 Endocrinology and Nephrology, CHU de Québec Research Center—Université Laval, Quebec City, QC G1V 4G2, Canada; atena.mahdavi.1@ulaval.ca; 2 Laboratory of Molecular Pharmacology, CHU de Québec Research Center—Université Laval, Quebec City, QC G1V 4G2, Canada; jocelyn.trottier@crchudequebec.ulaval.ca (J.T.); olivier.barbier@crchudequebec.ulaval.ca (O.B.); 3 Faculty of Pharmacy, Université Laval, Quebec City, QC G1V 4G2, Canada; 4 Department of Molecular Biology, Medical Biochemistry, and Pathology, Université Laval, Quebec City, QC G1V 4G2, Canada; 5 Department of Kinesiology, Faculty of Medicine, Université Laval, Quebec City, QC G1V 4G2, Canada

**Keywords:** dairy intake, bile acids precursor, serum proteins, cholesterol clearance, hyperinsulinemia

## Abstract

Bile acids regulate glucose homeostasis and lipid metabolism. Further, the levels of bile acids can be influenced by the intake of dairy products. Although the serum proteome can provide information on the biological pathways associated with different metabolites, it is unknown whether the intake of dairy modifies such associations between bile acids and the proteome. The objectives of this study were to examine plasma bile acid profiles, find the correlations between bile acids and lipid as well as glycemic markers, and to uncover the correlation between bile acids and proteins after high dairy (HD) and adequate dairy (AD) intake among 25 overweight individuals with hyperinsulinemia. In this randomized crossover-trial study, hyperinsulinemia adults were randomized to both HD (≥4 servings/day) and AD (≤2 servings/day) for 6 weeks. Measurements and analyses were performed on before- as well as after- AD and HD conditions. The results indicated that plasma 7α-hydroxy-4-cholesten-3-one (7AC4) increased after HD in comparison with before HD intake (*p* = 0.03). After adjusting for BMI, age, and sex, 7AC4 positively correlated with triglyceride levels in the pre-AD (r = 0.44; *p* = 0.03) and post-HD (r = 0.42; *p* = 0.04). Further, 7AC4 correlated positively with proteins associated with high-density lipoprotein particle remodeling pathway and reverse cholesterol transport only after HD consumption. Thus, the consumption of higher dairy intake modifies the association between 7AC4—a biomarker for bile acid synthesis—and serum proteins involved in cholesterol clearance. Overall, higher dairy consumption may have a positive effect on cholesterol metabolism in subjects at risk of type 2 diabetes.

## 1. Introduction

Type 2 diabetes (T2D) is a chronic medical condition characterized by elevated levels of blood sugar resulting from the body’s inability to effectively use insulin [1]. Hyperinsulinemia is considered to be an important risk factor for the development of T2D and is a condition in which chronic exposure to elevated insulin levels often contributes to insulin resistance, ultimately leading to T2D [1]. Individuals with T2D often exhibit dyslipidemia, which is an abnormal lipid profile. This typically includes elevated levels of triglycerides (TG) and low-density lipoprotein cholesterol (LDL-C), as well as decreased levels of high-density lipoprotein cholesterol (HDL-C). Recently, research has proposed that the accumulation of cholesterol in beta-cells, resulting from defective high-density lipoprotein (HDL) cholesterol and reduced cholesterol removal, leads to hyperglycemia, diminished insulin secretion, and the death of beta-cells [2]. On the other hand, elevated triglyceride levels can increase insulin resistance by promoting the accumulation of fatty acids within cells, inducing inflammation, and disrupting insulin signaling pathways [3]. These effects collectively contribute to impaired glucose metabolism and an increased risk of T2D. Bile acid levels have shown promise as potential biomarkers for T2D, as their altered metabolism and signaling have been correlated with insulin resistance, impaired glucose homeostasis, and lipid metabolism [4]. Furthermore, emerging research suggests that disruptions in bile acid signaling may contribute to the development of T2D [4,5].

Bile acids are amphiphilic molecules produced from cholesterol catabolism through a series of enzymatic reactions in the liver and they play important roles in emulsifying and solubilizing dietary fats in the small intestine. Bile acids are classified into primary and secondary bile acids. Primary bile acids, which include cholic acid and chenodeoxycholic acid, are synthesized in the liver and secreted into the bile ducts. Once in the intestine, primary bile acids are metabolized by gut bacteria into secondary bile acids such as deoxycholic acid and lithocholic acid [6]. Secondary bile acids, as well as cholate, chenodeoxycholate, and deoxycholate, are the most abundant bile acids in humans that influence metabolic processes including lipid and glucose metabolism [7,8]. There are two main pathways involved in bile acid biosynthesis: the classic pathway (neutral) and the alternative pathway (acidic). The classic pathway, which is also the most important in liver cells, is initiated by the microsomal enzyme cholesterol 7α-hydroxylase (CYP7A1), which converts cholesterol into 7α-hydroxycholesterol. Subsequently, 7α-hydroxycholesterol is transformed into 7α-hydroxy-4-cholesten-3-one (7αC4) [8]. 7αC4 serves as a common serum biomarker, reflecting bile acid synthesis, and acts as the precursor for the two primary bile acids synthesized in the human liver, namely cholic acid and chenodeoxycholic acid [8]. Importantly, bile acids can activate signaling pathways that modify lipid and glucose profiles [7].

Dietary intake can alter bile acid metabolism leading to a reduced risk of T2D [8]. Fermented dairy like yogurt, milk drinks, and kefir boost gut bacteria and short-chain fatty acid production that aid in lowering cholesterol [9]. The effect of bacteria occurs through improved bile acid deconjugation and reduced cholesterol in the circulation [10]. Moreover, bile acids produced by the microbiota regulate different aspects of immunity, including the induction of inflammatory genes to the recruitment of innate and adaptive immune cells [10,11,12]. Additionally, consuming high-fat dairy items can lead to a rise in the release of bile acids from the gallbladder, as a greater amount of bile is required to digest and absorb dietary fat. The intake of calcium through dairy consumption is also linked to bile acid levels. Previous study found that milk product-derived calcium contributes to the increased excretion of secondary bile acids in feces [13]. Therefore, the consumption of dairy products can potentially modify the regulation of bile acid metabolism.

Serum proteins play a crucial role in various physiological processes and can serve as important biomarkers for diagnosing and monitoring certain medical conditions [14]. Proteomics analysis can thus potentially reveal the association between bile acids and serum proteins, providing valuable information about their roles in metabolism, signaling pathways, and potential implications for human health. Importantly, no study has reported the association of specific blood proteins to the levels of bile acids in humans. In the present study, the bile acid profiles after high dairy products intake (HD, with at least 4 servings per day) compared to an adequate dairy product intake (AD, with no more than 2 servings per day) were measured. Additionally, the correlations between bile acids and lipid markers in subjects with hyperinsulinemia were examined. Finally, an untargeted proteomic-based mass spectrometry approach was used to uncover the associations between different serum proteins and serum bile acid levels among overweight individuals with hyperinsulinemia.

## 2. Materials and Methods

A factorial design was conducted for a cross-over randomized controlled trial. The recruitment of participants took place at the Centre Hospitalier Universitaire (CHU) de Québec-Université Laval Research Center between February 2017 and July 2018. A total of twenty-seven hyperinsulinemic adults (comprising 19 men and 8 women, with an average BMI of 31.3 ± 3.3 kg/m^2^ and an average age of 55 ± 14 years) were selected based on specific criteria.

These criteria included having fasting insulin levels above 90 pmol/L, fasting glucose levels below 7.0 mmol/L, and glycated hemoglobin (HbA1c) levels below 6.5%. The recruitment process involved using advertisements or email lists. The research received ethical approval from the ethics committee of the CHU de Québec-Université Laval Research Center (2017–3228 and 2022–6092) and was carried out following the ethical guidelines established in the Declaration of Helsinki. All patients were informed with comprehensive information regarding the trial’s objectives and procedures, and the participants were each provided with signed informed consent forms. Furthermore, the trial was officially registered on ClinicalTrials.gov (accessed on 24 September 2023) under the identifier NCT02961179.

### 2.1. Dietary Intervention

Eligible subjects were randomly allocated to either the high dairy product consumption condition (HD), instructed to consume at least four servings per day following the serving sizes recommended by Canada’s Food Guide for Healthy Eating (2007) [15] where a serving is defined as one cup (250 mL) of milk, ¾ cup (175 g) of yogurt or kefir, or 1.5 ounces (50 g) of cheese. Consumption of dairy products included milk, yogurt, cheese, kefir, and cream (≤15% fat content). Ice cream was also allowed, with one serving equivalent to ½ cup (125 mL) but was restricted to a maximum of three servings for each week. However, certain items like butter, whipped cream, cream exceeding 15% fat content, altered milk products, milk alternatives, and related products were excluded from the daily portion calculation. In contrast, adequate dairy intake (AD) was limited to no more than two servings per day.

Each intervention period lasted for 6 weeks, followed by a 6-week washout period, after which subjects were switched to the other intervention. A previous meta-analysis involving 38 studies revealed a range of exposure durations from 1 to 48 weeks. Notably, 13 of these studies had intervention periods lasting 6 weeks or less [16]. The choice to utilize a six-week intervention period was carefully considered to enhance participant adherence in a real-world, free-living environment. The dietary intake of subjects was assessed during each visit using a validated food frequency questionnaire (FFQ) consisting of 91 items and 33 sub-questions. The FFQ was administered through a web platform directly linked to the Nutrition Data System for Research [17]. To calculate the dietary intake, the Canadian nutrient file of 2015 was utilized [18]. Metabolomics analyses were also performed to confirm the compliance to HD and AD phases [19]. Results show higher serum pentadecanoic acid and heptadecanoic acid, both known biomarkers for dairy intake, after HD phase [19].

### 2.2. Anthropometric Assessments

Anthropometric measurements, including weight (kg) and height (cm), were taken during the study. Weight was recorded using a precise professional scale (Health O Meter Professional, Sunbeam products, Inc., Boca Raton, FL, USA) with an accuracy of 0.1 kg, and height was measured with a wall-mounted stadiometer (The Easy-Glide Bearing Stadiometer, Perspective Enterprises) accurate to 1 mm. During these measurements, participants were instructed to wear light clothing and no shoes. Body Mass Index (BMI) was calculated by dividing the weight in kilograms by the square of the height in meters.

### 2.3. Clinical Measurements

The study involved a total of four visits, occurring both at the start and end of each intervention period, with six-week intervals between them. Fasting blood glucose (FBG) levels were determined using hexokinase assays [14], while fasting insulin levels were assessed through chemiluminescence immunoassay [15]. The calculation of the Homeostatic Model Assessment of Insulin Resistance (HOMA-IR) was based on the following formula: [insulin (pmol/L) × glucose (mmol/L)]/135 [20]. Enzymatic assays was used to measure plasma total cholesterol (TC) and triglyceride (TG) concentrations [21,22]. The high-density lipoprotein (HDL-C) fraction was received after precipitation of very low-density lipoprotein and LDL particles in the infranatant with heparin manganese chloride [23]. Low-density lipoprotein cholesterol (LDL-C) was calculated using the Friedewald formula [24].

### 2.4. Bile Acids Measurement

Fasting plasma samples were obtained in the morning from each subject at four time points (before and after AD and HD) to standardize the conditions and reduce the influence of circadian rhythms. These samples were used to analyze bile acids.

Specifically, samples were analyzed using a LC–MS/MS system, employing a method adapted from Daniel N et al. [25]. Solid-phase extraction (SPE) was performed by adding 2 mL of a 0.1% (*w*/*v*) formic acid solution and 50 μL of internal standards (i.e., the deuterated Bas, d4-CDCA, d4-CA, d4-LCA, d4-DCA, d5-TCA, and d4-GCA) to 50 μL of plasma. Analytical standards underwent the same treatment with a 1:1 dilution using 50 μL of adsorbed plasma, generating calibration equations. Strata-X 60 mg SPE columns (Phenomenex, Torrance, CA, USA) were conditioned with 1 mL of MeOH and 2 mL of H_2_O-0.1% formic acid. After applying the samples, columns were washed successively with 2 mL of H_2_O and 2 mL of H_2_O:MeOH (80:20) containing 0.1% formic acid under negative pressure. Bile acids were eluted with 2 mL of MeOH. Eluates were fully evaporated at 35 °C under N_2_ and reconstituted in 100 μL of H_2_O:MeOH (50:50). For analysis, 1 microlitre of samples or calibration standards were injected into the LC–MS/MS system.

All analytes were quantified using tandem mass spectrometry with an API6500 instrument equipped with an electrospray ionization source (Applied Biosystems, Concord, ON, Canada). Chromatographic separation employed a Nexera ultra-high-pressure liquid chromatography (UHPLC) instrument (Shimadzu Scientific Instruments, Columbia, MD, USA) and a 150 × 2.1 mm Poroshell 120 EC-C18; 2.7 μm particles column (Agilent, Santa Clara, CA, USA). The chromatographic system utilized a 5 mM gradient of water, ammonium acetate at pH 7.7, and acetonitrile at a flow rate of 300 μL/min. The source temperature was set at 500 °C.

### 2.5. Identification and Quantifications of Serum Proteins by Mass Spectrometry

The identification and quantifications of serum proteins from our cohort were performed exactly as previously described [26]. Briefly, every sample, which included a 0.5X concentration of iRT peptides from Biognosys in Schlieren, Switzerland, underwent nanoLC-MS/MS analysis using a Dionex UltiMate 3000 nanoRSLC chromatography system (Thermo Fisher Scientific, San Jose, CA, USA) connected to an Orbitrap Fusion mass spectrometer (Thermo Fisher Scientific) with a nanoelectrospray ion source. Peptides were trapped at 20 μL/min in loading solvent (2% I/0.05% TFA) on a 5 mm × 300 μm C18 PepMap cartridge pre-column (Thermo Fisher Scientific) for 5 min. Afterward, the preceding column was connected to a PepMap Acclaim C18 column measuring 50 cm in length and 75 µm in internal diameter, provided by Thermo Fisher Scientific. Peptides were then separated by a gradual increase in solvent B (where A is composed of 0.1% formic acid and B contains 80% isopropanol with 0.1% formic acid) over 90 min, with a flow rate of 300 nanoliters per minute.

The mass spectrometry data were obtained using the All-Encompassing Data Collection (DIA) mode with Version of Thermo Xcalibur software 4.3.73.11. Internal calibration was conducted utilizing the lock mass function with the siloxane ion with a mass-to-charge ratio of 445.12003. The spray voltage was adjusted to 2100 V, while maintaining a constant heated capillary temperature of 275 °C. Full scan mass spectra were recorded in the Orbitrap, ranging from 350 to 1000 *m*/*z*, at a resolution of 60,000, with an AGC target of 4 × 10^5^ and an IT of 50 ms. Following each MS scan, 75 fragmentation MS/MS spectra with a width of 8 *m*/*z* were acquired, covering the range of 350 to 950 *m*/*z*. Quadrupole isolation was employed to select precursor ions, which were then subjected to Higher Energy Collision-Induced Dissociation (HCD) with a collision energy of 35 percent. The ensuing fragment ions were identified in the Orbitrap at a resolution of 15,000 (200–1500 *m*/*z*), with a target AGC of 5 × 10^4^, and an ion time (IT) of 22 ms.

The initial step involved converting the raw data files into mzML format using MSConvert version 3.0.21188. Signal extraction and quantification were then conducted through DIA-NN version 1.8 [23]. A Homo Sapiens fasta file from UniProt Reference Proteome (Proteome ID UP000005640, July 2020 release, containing 63,807 entries) was utilized in this analysis. The approach employed deep learning-based spectra. Trypsin/P enzyme cleavage was chosen, allowing for a maximum of two missed cleavages and permitting one variable modification (N-terminal Methionine excision and Methionine oxidation; Carbamidomethylation of Cysteine was considered a fixed modification) per peptide. Peptides ranging from 7 to 30 amino acids were included in the analysis. Precursor ions with a charge state of 2 + to 4 + and within the *m*/*z* range of 350 to 950 were selectively retained, while the fragment mass range was set to 200–1500 *m*/*z*. Match Between Runs (MBR) functionality was activated, and all other parameters were maintained at their default settings. The complete mass spectrometry data have been openly shared on the ProteomeXchange repository (https://www.proteomexchange.org) (accessed on 12 December 2022) under the unique identifier PXD035386.

### 2.6. Statistical Analyses

Statistical analysis was conducted using SPSS version 26 for macOS with the significance level set to <0.05. The normality of the data distribution was assessed using the Shapiro–Wilk test. In cases where the variables did not meet parametric criteria, a non-parametric approach was employed such as the Wilcoxon signed-rank test for paired data and the Mann–Whitney test for independent data. The comparisons of baseline and endpoints parameters, including anthropometric, lipid profile (including HD, TC, LDL and TG), glycemic profile (comprising FBG, fasting insulin, and HOMA-IR), bile acids profile, and dietary intakes, both within and between the groups, were conducted using paired *t*-tests and independent tests for variables meeting parametric assumptions. In addition, Spearman correlation was applied to investigate the relationship between serum bile acid levels and lipid and glycemic profiles within each condition after adjusting for age, sex, and BMI. Furthermore, this analysis was utilized to explore the quantitative association between serum protein levels and bile acid levels within the same groups.

## 3. Results

### 3.1. Characteristics of Participants

The research involved twenty-seven participants, comprising eight women and nineteen men. Among these, serum proteomic profiles were gathered from twenty-five individuals, consisting of eighteen men and seven women. Table 1 displays the characteristics of these volunteers, including an average age and BMI of 54 ± 14 years and 31.2 ± 3.0, respectively. All subjects exhibited fasting hyperinsulinemia with HOMA-IR > 2 or FBG more than 6.1 mmol/L. When comparing dairy product consumption after the AD and HD phases, it was noted that the average intake was 2.27 ± 1.22 servings per day and 5.95 ± 1.79 servings per day, respectively (*p* < 0.001). No differences in anthropometric and biochemical measurements were observed between the HD and AD groups at the outset (0 week). Initial results at 0 weeks revealed a decrease in TC (*p* = 0.04) following AD in comparison with before AD intake. However, no change in total cholesterol was observed between before and after HD intake. After post-HD consumption, there were increases in fasting insulin, HOMA-IR, and triglycerides compared to the pre-HD condition (*p* = 0.02, 0.02, 0.05, respectively). Conversely, no differences were observed for these parameters between before and after AD condition with *p* = 0.23, 0.96, and 0.60, respectively. Nonetheless, despite the changes observed between before- and after-HD conditions, the levels of fasting insulin, HOMA-IR, TG, LDL, HDL, and the ratios of total cholesterol/HDL and LDL/HDL remained stable after HD consumption compared to after AD consumption.

### 3.2. Dietary Intakes

The average dietary consumption in the pre-AD, pre-HD, and post-AD groups showed no significant differences. However, certain differences were observed between post-HD and post-AD consumption. On one hand, total intakes of protein, calcium, saturated fat (SFA), animal protein, % calories from protein, and % calories from SFA were higher in the post-HD group (*p* = 0.007, <0.001, 0.01, <0.001, 0.04, 0.02, respectively). On the other hand, % calories from monounsaturated fat (MUFA) and polyunsaturated fat (PUFA) were lower in the post-HD group (*p* = 0.03, <0.001). Furthermore, when comparing the differences between before and after HD, variations were noted in the averages of energy intake, protein consumption, cholesterol intake, SFA intake, calcium intake docosapentaenoic acid intake (DPA), total trans-fat (TFA) intake, as well as the percentage of calories from protein, SFA, PUFA, and MUFA (*p* < 0.05 for all these factors as shown in Table 1). The data presented in Table 1 are derived from a secondary analysis of the original dataset. The detailed description can be found in our previous article [26]. Further, calcium intakes before as well as after AD and HD consumption were added to the table.

### 3.3. Plasma Bile Acid Levels

Table 2 reveals that there were no differences in the mean individual bile acids before and after dairy product consumption, except for the bile acid precursor 7αC4, which increased after post-HD compared to pre-HD (*p* = 0.03). Therefore, the current study focused on 7αC4 and its correlation with lipids, glycemic markers, and serum proteins as well.

### 3.4. Serum Proteomic Levels

A total of 231 serum proteins were measured through mass spectrometry analyses in all samples and no difference was detected between before- and after- AD and HD conditions as described previously for this cohort [26] (Supplementary Table S1).

### 3.5. Correlations between Total or Individual Serum Bile Acid Levels and Glycemic or Lipid Profiles in AD vs. HD Groups after Adjustments for Age, Sex and BMI

After adjusting for BMI, age, and sex [27,28,29], only 7αC4 correlated with triglyceride levels (r = 0.44, *p* = 0.03; r = 0.42, *p* = 0.04) in the before-AD and -HD conditions, respectively (Table 3).

### 3.6. Correlations between Serum 7αC4 and Serum Proteins before and after AD and HD Conditions

Spearman analysis was performed to detect proteins correlating to serum 7αC4 levels in separate conditions (before and after AD and HD intake). Table 4 shows the proteins that exhibited positive and negative correlations with 7αC4 in each condition. Specifically, in the before- and after- AD, and HD conditions, seventeen, eight, seven, and thirteen proteins exhibited a positive correlation with 7αC4. Furthermore, eight, six, twelve, and six proteins exhibited negative correlations with 7αC4. To gain insight into the biological significance, the protein sets were examined through the Database for Annotation, Visualization, and Integrated Discovery (DAVID) software [30]. In pre-AD, the proteins that correlated positively to 7αC4 were associated with complement activation, cholesterol, and lipoprotein metabolic processes, while no biological processes were uncovered with the proteins that correlated negatively with 7αC4. The biological processes that correlated positively with serum 7αC4 level in post-AD included several aspects of the immune response including adaptive immune response, the classical complement activation pathway, the B cell receptor signaling pathway, immunoglobin production, and phagocytosis recognition and engulfment. The proteins that correlated negatively with 7αC4 in the post-AD conditions were involved in the negative regulation of peptidase activity. In the pre-HD conditions, only proteins associated with the modulation of the immune response exhibited an inverse correlation with the presence of 7αC4. Finally, in the post-HD conditions, several proteins that correlated positively with 7αC4 were involved with HDL lipoprotein particle remodeling and reverse cholesterol transport, while proteins associated with the complement activation pathways were inversely correlated with serum 7αC4 levels (Table 5).

## 4. Discussion

This work is the first cross-over study which reports the association between specific serum proteins and individual bile acids in hyperinsulinemia subjects after the consumption of adequate or high dairy intake. The main finding is that higher consumption of dairy products modifies the association between 7αC4—a biomarker for bile acid synthesis—and serum proteins involved in the immune system and lipid metabolism. Importantly, 7αC4 correlated positively with proteins associated with pathways for remodeling high-density lipoprotein particles and facilitating the reverse transport of cholesterol exclusively in the post-HD condition.

Specifically, the post-HD group exhibited higher dietary cholesterol, SFA, DPA, TFA, MUFA, and PUFA levels compared to the pre-HD, pre-AD and post-AD groups [19,26]. Accordingly, another investigation demonstrated that incorporating dairy products into the diet altered fat intake, especially SFA and cholesterol [31]. The consumption of higher-fat dairy products might increase the amount of bile needed; therefore, potentially influencing bile acid metabolism [32]. Indeed, changes in fat and cholesterol intake can modify the synthesis, regulation, and recycling of bile acids, which, in turn, affect fat digestion, absorption, and cholesterol metabolism in the body [13]. Further, Govers et al. [33] indicated that calcium in milk products also increases fecal excretion of secondary bile acids. The authors suggested that the increase in fecal bile acid excretion may be due to increased bile acid synthesis and secretion in response to calcium intake [34]. Cheese, a high-fat dairy product, is believed to be a contributor to this effect due to its calcium or calcium phosphate complexes binding to bile acids in the intestines [33]. This results in the removal of bile acids from the enterohepatic cycle, requiring the liver to generate more bile acids from cholesterol. However, not all studies have observed an increase in fecal bile acid excretion with calcium in cheese [34]. Inconsistencies observed in these studies could be related to the design of the study, the type of intervention (adequate or high dairy intake), and the subjects’ characteristics involved. Nevertheless, the current results are consistent with the previous research suggesting that high dairy product consumption can affect dietary fat and calcium intake potentially leading to an increased demand for precursors of bile acids.

Interestingly, a positive correlation was observed between 7αC4 and triglyceride levels in both the pre-AD and post-HD conditions. Concurrently, 7αC4 exhibited a positive correlation with proteins involved in cholesterol and lipoprotein metabolic processes in the pre-AD condition. The proteins associated with these biological processes were APOC3, APOC1, and APOL1. These proteins are known components of lipoproteins responsible for transporting lipids, such as cholesterol and triglycerides, in the bloodstream. APOC3 and APOC1 play central roles in HDL metabolism [35]. Dysregulation of lipid metabolism and abnormalities in lipoprotein levels and activities are well-recognized risk factors for cardiovascular diseases associated with T2D [36]. APOL1, in return, may contribute to the body’s inflammatory response with inflammatory signaling molecules like interferon-γ (IFN), and tumor necrosis factor-α (TNF-α). Accordingly, a positive correlation emerged between 7αC4 and proteins involved in the classical pathway of complement activation in hyperinsulinemic subjects with AD intake. Complement activation plays a pivotal role in supporting the immune response, fostering inflammation, and aiding in the elimination of pathogens and cellular debris [37]. This pathway can contribute to effective immune responses [38]. Furthermore, there was a positive correlation observed between 7αC4 and proteins belonging to the class of immunoglobulin heavy chain, which are associated with various immune system responses. In addition, the B cell receptor signaling pathway is a crucial process that allows B cells to detect and respond to antigens [39]. B cells play a critical role in adaptive immunity by producing antibodies that can neutralize or eliminate pathogens or abnormal cells [39]. The mechanism of correlation between 7αC4 and immune system responses is still unknown. Nonetheless, bile acids have been shown to exert a modulatory effect on inflammation by reducing the expression of pro-inflammatory cytokines and influencing immune cell responses [40,41]. Importantly, chronic, low-grade inflammation is a prevalent characteristic of numerous chronic metabolic disorders such as obesity and T2D [42,43].

Further, a positive association between 7αC4 and proteins involved in high-density lipoprotein particle remodeling and reverse cholesterol transport were observed after high dairy intake (post-HD). Reverse cholesterol transport is a critical process that involves the transportation of excess cholesterol from peripheral tissues, including arterial walls, back to the liver for eventual elimination from the body. This process is largely facilitated by HDL, which play a central role in collecting cholesterol from tissues and transporting it to the liver [44]. HDL particles undergo dynamic changes and remodeling during this process, transitioning from nascent discoidal structures to mature spherical forms with a cholesterol-rich core [44]. These mechanisms can reduce the risk of cardiovascular complications in T2D subjects. There is little information about 7αC4 and cholesterol clearance, but there is some evidence indicating that reverse cholesterol transport is active in the process of removal of excess cholesterol from vascular plaques and the transport of this cholesterol to the liver for degradation through bile acids [45]. As such, the current study demonstrates that a higher consumption of dairy products leads to an increase in the precursor of bile acids synthesis and its association with processes that enhance cholesterol clearance with potential beneficial effects for the health of overweight hyperinsulinemia subjects.

In addition to the association of 7αC4 with HDL lipoprotein particle remodeling and cholesterol clearance processes after consumption of high dairy intake, 7αC4 negatively correlated with serum proteins involved in complement activation and the killing of cells from other organisms. Specifically, proteins involved in biological processes like CFD, C9, and C8B correlated negatively with 7αC4. Notably, the occurrence of oxidative harm to cell membranes in various organs has the potential to increase the initiation of the classical complement pathway. The complement system not only enhances the body’s ability to clear pathogens and stimulate inflammation, but also helps in the removal of damaged cells [39]. The high dairy intake condition down modulates the 7αC4 association with certain aspects of the innate immune system which may reflect a better regulation of a low but chronic inflammatory response. Thus, bile acids are recognized not just for their roles in digestion and absorption, but also for their involvement in the modulation of immune responses [46].

There are several limitations in our study. First, the small sample size may have limited the statistical power and sensitivity, potentially indicating our ability to detect correlations between different serum bile acids and various proteins. Second, the study participants were overweight/obese adults with hyperinsulinemia, and it remains uncertain whether the findings are representative of other chronic conditions that may impact dyslipidemia, oxidative stress, and chronic inflammation. Third, the present study focused only on 7αC4 as other bile acids did not exhibit changes across different conditions. Another limitation pertains to the short study duration. Further, changes in bile acids may require a more extended period of higher dairy consumption to become significant. Thus, extending the period of higher dairy intake may show more comprehensive insights into the beneficial effects of dairy products on the pathophysiology of hyperinsulinemia and the relationship between bile acids and serum proteins. The potential for residual confounding factors is possible. However, the current study design was a randomized controlled trial which reduces potential for confounding by generating groups that are comparable with respect to known and unknown confounding variables.

## 5. Conclusions

In the post-HD condition, the biomarker 7αC4, indicative of bile acid synthesis, exhibited a correlation with proteins associated with the pathway of remodeling high-density lipoprotein particles and the reversal of cholesterol transport. Overall, even if higher dairy product consumption includes higher cholesterol and SFA intake, their consumption may induce a positive effect on cholesterol metabolism in individuals at risk of T2D.

## Figures and Tables

**Table 1:** Features and dietary consumption of individuals with hyperinsulinemia in AD and HD consumption.

Variables	AD		*p* *	HD		*p* *	*p* **	*p* ***
	(Mean ± SD)			(Mean ± SD)				
	0 Week	6 Weeks		0 Week	6 Weeks			
Body weight (kg)	90.44 ± 15.60	90.31 ± 15.72	0.73	89.85 ± 15.43	90.27 ± 15.43	0.1	0.17	0.99
BMI (kg/m^2^)	31.37 ± 3.39	31.32 ± 3.39	0.85	31.15 ± 3.18	31.30 ± 3.25	0.1	0.96	0.96
HDL (mmol/L)	1.12 ± 0.23	1.10 ± 0.24	0.43	1.12 ± 0.26	1.09 ± 0.27	0.23	0.92	0.87
LDL (mmol/L)	2.72 ± 0.85	2.60 ± 0.90	0.05	2.71 ± 0.86	2.60 ± 0.99	0.33	0.95	0.97
Cholesterol total (mmol/L)	4.63 ± 0.96	4.46 ± 0.95	0.04	4.57 ± 1.00	4.49 ± 1.16	0.56	0.93	0.93
Ratio cholesterol total/HDL	4.37 ± 1.69	4.26 ± 1.47	0.36	4.30 ± 1.66	4.38 ± 1.98	0.54	0.91	0.91
Ratio LDL/HDL	2.55 ± 1.01	2.44 ± 0.93	0.16	2.57 ± 1.12	2.54 ± 1.25	0.9	0.8	0.8
Fasting blood glucose (mmol/L)	5.26 ± 0.48	5.24 ± 0.55	0.85	5.19 ± 0.45	5.32 ± 0.59	0.35	0.44	0.7
Fasting insulin (pmol/L)	119.95 ± 57.25	127.43 ± 74.07	0.23	113.54 ± 54.69	133.34 ± 73.44	0.02	0.45	0.67
Insulin resistance, HOMAIR	4.95 ± 2.59	5.24 ± 3.92	0.96	4.43 ± 2.35	5.26 ± 3.47	0.02	0.73	0.83
TG (mmol/L)	1.72 ± 1.13	1.68 ± 1.15	0.60	1.57 ± 0.95	1.73 ± 1.01	0.05	0.5	0.57
Dietary Intake								
Dairy products (servings/d)	2.95 ± 2.16	2.27 ± 1.21	0.20	2.5 ± 1.74	5.94 ± 1.78	<0.001	0.24	<0.001
Calcium intake (mg/d)	1388.16 ± 765.75	1155.20 ± 405.66	0.18	1236.04 ± 628.14	2228.36 ± 588.05	<0.001	0.42	<0.001
Total energy intake (kcal/d)	2355.27 ± 1094.34	2100.94 ± 792.24	0.28	2174.68 ± 1054.06	2493.83 ± 896.82	0.03	0.28	0.11
Total carbohydrate intake (g/d)	263.35 ± 116.37	235.96 ± 97.40	0.08	246.27 ± 128.44	278.69 ± 111.75	0.05	0.32	0.16
Total protein intake (g/d)	104.23 ± 50.37	92.66 ± 33.57	0.28	94.58 ± 42.81	119.40 ± 33.83	<0.001	0.31	0.007
Total fat intake (g/d)	96.17 ± 52.64	83.87 ± 33.83	0.43	88.81 ± 46.53	97.71 ± 39.20	0.21	0.3	0.19
Cholesterol intake (mg/d)	316.98 ± 176.86	281.74 ± 110.82	0.51	278.61 ± 129.39	332.05 ± 140.60	0.04	0.24	0.17
Total Saturated Fat (SFA) intake (g/d)	34.5 ± 23.76	28.13 ± 12.40	0.41	31.05 ± 18.15	39.42 ± 17.10	0.01	0.32	0.01
Total Monounsaturated Fat (MUFA) intake (g/d)	38 ± 19.81	33.86 ± 14.22	0.29	35.11 ± 18.41	36.54 ± 15.04	0.61	0.61	0.52
Total Polyunsaturated Fat (PUFA) intake (g/d)	16.44 ± 6.85	15.45 ± 5.86	0.32	15.87 ± 8.07	14.78 ± 5.90	0.4	0.23	0.69
Arachidonic acid (AA) intake (g/d)	0.15 ± 0.08	0.14 ± 0.06	0.84	0.14 ± 0.07	0.13 ± 0.06	0.17	0.29	0.29
Eicosapentaenoic acid (EPA) intake (g/d)	0.22 ± 0.30	0.24 ± 0.30	0.86	0.19 ± 0.24	0.19 ± 0.29	0.07	0.55	0.17
Docosapentaenoic acid (DPA) intake (g/d)	0.04 ± 0.04	0.04 ± 0.03	0.96	0.04 ± 0.03	0.03 ± 0.03	0.01	0.82	0.1
Docosahexaenoic acid (DHA) intake (g/d)	0.27 ± 0.28	0.28 ± 0.26	0.8	0.24 ± 0.22	0.22 ± 0.24	0.07	0.88	0.17
Total Trans-Fat intake (g/d)	4.00 ± 2.61	3.43 ± 1.97	0.18	3.64 ± 2.89	3.92 ± 2.37	0.03	0.82	0.43
% calories from protein	18.05 ± 4.14	18.08 ± 4.11	0.82	17.97 ± 3.12	19.76 ± 3.13	<0.001	0.15	0.04
% calories from carbohydrate	44.81 ± 6.88	44.83 ± 8.12	0.88	45.55 ± 7.65	44.66 ± 6.58	0.41	0.78	0.61
% calories from fat	36.45 ± 4.96	35.97 ± 5.92	0.61	36.37 ± 6.11	34.98 ± 4.80	0.19	0.67	0.52
% calories from SFA	12.7 ± 2.97	11.99 ± 3.10	0.25	12.45 ± 3.19	14.15 ± 3.36	0.003	0.45	0.02
% calories from MUFA	14.52 ± 2.14	14.47 ± 2.56	0.89	14.41 ± 2.54	13.04 ± 1.97	0.01	0.49	0.03
% calories from PUFA	6.44 ± 1.45	6.73 ± 1.37	0.16	6.71 ± 1.88	5.29 ± 0.84	<0.001	0.29	<0.001

The number of participants: 25. Information displayed as the mean ± standard deviation. * The paired *t*-test and Wilcoxon test were used to comparison within the groups (pre-AD vs. post AD/pre-HD vs. post-HD). *** The independent *t*-test and Mann–Whitney test were used to compare between the groups (post-AD vs. post-HD). ** Assessment of the contrast between the groups before adequate dairy intake (pre-AD) and before high dairy intake (pre-HD). Significance was defined as a *p*-value of less than 0.05.

**Table 2:** Characteristics of bile acids during high dairy or adequate dairy consumption.

	AD		*p* *	HD		*p* *	*p* **	*p* ***
Parameters	(Mean ± SD)			(Mean ± SD)				
	0 Week	6 Weeks		0 Week	6 Weeks			
CA (Cholic acid)	169.26 ± 276.87	197.87 ± 303.71	0.47	172.35 ± 236.13	196.91 ± 344 72	0.94	0.95	0.65
CDCA (Chenodeoxycholic acid)	234.33 ± 373.26	227.56 ± 298.77	0.61	230.77 ± 337.40	226.58 ± 342.57	0.58	0.63	0.73
DCA (Deoxycholic acid)	257.91 ± 206.98	283.62 ± 174.13	0.56	304.71 ± 214.47	321.50 ± 251	0.79	0.35	0.93
HDCA (Hyodeoxycholic acid)	1.12 ± 1.01	1.37 ± 1.56	0.42	1.85 ± 2.8	0.99 ± 1.45	0.08	0.50	0.30
UDCA (Ursodeoxycholic acid)	68.42 ± 117.56	53.95 ± 75.39	0.46	52.78 ± 74.43	45.98 ± 55.01	0.66	0.65	0.98
HCA (Hyo-cholic acid)	7.57 ± 10.72	10.32 ± 15.21	0.12	9.77 ± 12.51	7.17 ± 8.01	0.21	0.25	0.72
LCA (lithocholic acid)	9.74 ± 7.58	10.47 ± 11.45	0.47	11.26 ± 7.33	11.15 ± 7.41	0.64	0.34	0.23
GCA (Glyco-cholic acid)	131.95 ± 136.58	98.71 ± 79.03	0.41	92.45 81.40	114.13 ± 147.78	0.29	0.18	0.85
GCDCA (Glyco-cheno-deoxycholic acid)	366.63 ± 303.73	283.05 ± 182.01	0.37	320.07 ± 193.92	327.84 ± 360.01	0.70	0.93	0.91
GDCA (Glyco-deoxycholic acid)	222.75 ± 335.45	183.75 ± 145.93	0.82	222.80 ± 254.63	183.85 ± 193.80	0.31	0.85	0.61
GUDCA (Glycoursodeoxycholic acid)	74.94 ± 87.63	62.13 ± 59.91	0.92	59.82 ± 49.49	68.09 ± 58.72	0.17	0.95	0.67
GLCA (Glycolithocholic acid)	7.87 ± 8.68	8.20 ± 9.13	0.81	10 ± 13.14	8.26 ± 8.29	0.31	0.49	0.40
TCA (Taurocholic acid)	42.99 ± 55.99	34.75 ± 54.17	0.37	25.79 ± 21.88	31.01 ± 38.80	0.56	0.64	0.85
TCDCA (Tauro-cheno-deoxycholic acid)	77.85 ± 98.18	59.90 ± 55.61	0.88	56.76 ± 48.28	59.94 68.93	0.70	0.62	0.89
TDCA (Tauroursodeoxycholic acid)	49.30 ± 103.17	34.91 ± 38.23	0.78	36.33 ± 49.47	31.61 ± 37.16	0.99	0.93	0.91
TUDCA (Tauroursodeoxycholic acid)	2.77 ± 3.34	2.53 2.22	0.39	1.95 ± 1.82	2 ± 1.87	0.71	0.66	0.28
TLCA (Taurolithocholic acid)	1.38 ± 2.5	1.27 ± 1.57	0.77	1.57 ± 2.51	1.02 ± 1.04	0.26	0.48	0.88
3dhLCA (3-dehydroxycholic acid)	0.49 ± 0.78	0.49 ± 0.78	1	0.43 ± 0.75	0.59 ± 1.04	0.36	0.76	0.93
7aC4_177 (7α-hydroxy-4-cholesten-3-one 177)	75.51 ± 53.88	67.94 ± 60.05	0.24	67.92 ± 53.44	92.2 ± 91.33	**0.03**	0.56	0.32
TOTAL Primary bile acids	1023.03 ± 828.74	900.87 ± 757.18	0.73	898.22 ± 562.36	956.44 ± 955.38	0.51	0.73	1
TOTAL Secondary bile acids	695.12 ± 614.26	640.73 ± 339.22	0.75	701.20 ± 472.49	673.50 ± 444.88	0.51	0.75	0.64
TOTAL CA	344.21 ± 306.98	331.34 ± 386.24	0.42	290.60 ± 233.22	342.06 ± 408.01	0.86	0.42	0.70
TOTAL CDCA	678.8 ± 550.95	569.52 ± 403.71	0.51	607.61 ± 371.31	614.37 ± 573.24	0.42	0.51	0.79

The number of participants: 25. Information displayed as the mean ± standard deviation. * The paired *t*-test and Wilcoxon test were used to comparison within the groups (pre-AD vs. post AD/pre-HD vs. post-HD). *** The independent *t*-test and Mann–Whitney test were used to comparison between the groups (post-AD vs. post-HD). ** Assessment of the contrast between the groups before adequate dairy intake (pre-AD) and before high dairy intake (pre-HD). Significance was defined as a *p*-value of less than 0.05. Total primary bile acids: cholic acid (CA), glycocholic acid (GCA), taurocholic acid (TCA), chenodeoxycholic acid (CDCA), glycochenodeoxycholic acid (GCDCA), aurochenodeoxycholic acid (TCDCA). Total secondary bile acids: Deoxycholic acid (DCA), ursodeoxycholic acid (UDCA), lithocholic acid (LCA), glycodeoxycholic acid (GDCA), glycoursodeoxycholic acid (GUDCA), glycolithocholic acid (GLCA), taurodeoxycholic acid (TDCA), Tauroursodeoxycholic acid (TUDCA), taurolithocholic acid (TLCA). Tota; CA: CA + GCA + TCA. TOTAL GDCA: CDCA + GCDCA + TCDCA. In bold: the only significant bile acids in the table.

**Table 3:** Correlation between 7αC4 with lipid and glycemic markers after adjustments for age, sex, and BMI.

	FBG (mmol/L)	Fasting Insulin (pmol/L)	HOMA-IR	HDL (mmol/L)	LDL (mmol/L)	TC (mmol/L)	Ratio TC/HDL	Ratio LDL/HDL	TG (mmol/L)
Pre-AD									
Correlation	−0.05	0.1	0.26	−0.26	0.21	0.36	0.38	0.31	0.44
Significant	0.78	0.61	0.20	0.21	0.30	0.07	0.06	0.12	0.03 *
Post-AD									
Correlation	−0.01	0.282	0.27	−0.12	0.14	0.26	0.24	0.14	0.25
Significant	0.93	0.18	0.19	0.57	0.49	0.20	0.24	0.49	0.22
Pre-HD									
Correlation	0.18	0.16	0.19	−0.19	0.22	0.33	0.28	0.23	0.32
Significant	0.38	0.43	0.37	0.35	0.28	0.11	0.17	0.27	0.12
Post-HD									
Correlation	0.01	0.28	0.2	−0.22	0.07	0.18	0.22	0.1	0.42
Significant	0.94	0.18	0.32	0.28	0.73	0.37	0.28	0.61	0.04 *

* Significant *p*-value (*p* < 0.05 for the Spearman correlation rho value).

**Table 4:** Proteins present in the serum showing Spearman correlations with 7αC4 in before and after dairy conditions.

		Correlations (Pre-AD) Rho	*p*-Value
SERPINA7	Serpin Family A Member 7	0.59	0.002
IGLV2-8	Immunoglobulin Lambda Variable 2–8	0.42	0.003
IGKV3-20	Immunoglobulin Kappa Variable 3–20	0.41	0.004
SERPINA6	Serpin Family A Member 6	0.55	0.004
KNG1	Kininogen 1	0.54	0.005
IGKV1-12	Immunoglobulin Lambda Variable 1–12	0.50	0.009
APOE	Apolipoprotein E	0.50	0.011
CFI	Complement factor I	0.49	0.013
APOC1	Apolipoprotein C1	0.48	0.014
LUM	Lumican	0.48	0.014
C3	Complement component 3	0.47	0.015
IGHV3-15	Immunoglobulin heavy variable 3–15	0.47	0.017
CNDP1	Beta-Ala-His dipeptidase	0.45	0.022
APOL1	Apolipoprotein L-1	0.43	0.029
TF	Transferrin	0.42	0.034
F10	coagulation factor X	0.42	0.036
CLU	Clusterin	0.42	0.036
IGFALS	Insulin like growth factor binding protein acid labile subunit	−0.41	0.038
IGHV6-1	Immunoglobulin heavy variable 6-1	−0.41	0.039
APOB	Apolipoprotein B	−0.41	0.039
AMBP	A1M (α1-microglobulin)/bikunin precursor	−0.53	0.006
ITIH3	Inter-alpha-trypsin inhibitor heavy chain 3	−0.49	0.012
EFEMP1	fibulin-like extracellular matrix protein 1	−0.46	0.016
GC	Guanine-cytosine content	−0.43	0.028
SHBG	Sex hormone binding globulin	−0.4	0.046
		Correlations (post-AD)	
IGHV3-15	Immunoglobulin heavy variable 3–15	0.53	0.006
IGKV3-20	Immunoglobulin Kappa Variable 3–20	0.43	0.03
IGKV1-12	Immunoglobulin Kappa Variable 1–12	0.42	0.033
IGKV1D-16	Immunoglobulin Kappa Variable 1D-16	0.42	0.033
IGHV3-23	Immunoglobulin heavy variable 3–23	0.42	0.036
IGHG1	Immunoglobulin Heavy Constant Gamma 1	0.40	0.045
IGKV1-16	Immunoglobulin Kappa Variable 1–16	0.39	0.049
ECM1	Extracellular matrix protein 1	0.39	0.05
CA1	Carbonic anhydrase 1	−0.46	0.019
HBD	Hemoglobin Subunit Delta	−0.39	0.003
AMBP	A1M (α1-microglobulin)/bikunin precursor	−0.48	0.014
APOA4	Apolipoprotein A4	−0.57	0.025
AZGP1	Alpha-2-Glycoprotein 1, Zinc-Binding	−0.39	0.048
ITIH3	Inter-alpha-trypsin inhibitor heavy chain 3	−0.46	0.049
ITIH4	Inter-alpha-trypsin inhibitor heavy chain 4		
		Correlation (pre-HD)	
APOC3	Apolipoprotein C3	0.57	0.003
C1R	Complement C1r subcomponent	0.48	0.015
TF	Transferrin	0.48	0.015
IGKV1-16	Immunoglobulin Kappa Variable 1–16	0.43	0.031
CFI	Complement factor I	0.42	0.033
IGFBP3	Insulin-like growth factor (IGF)-binding protein-3	0.41	0.039
PCYOX1	Prenylcysteine Oxidase 1	0.39	0.049
FGA	Fibrinogen alpha chain	−0.47	0.017
AMBP	A1M (α1-microglobulin)/bikunin precursor	−0.60	0.001
GC	Guanine-cytosine content	−0.58	0.002
SHBG	Sex hormone binding globulin	−0.59	0.002
CLEC3B	C-Type Lectin Domain Family 3 Member B	−0.55	0.004
C8B	Complement C8 Beta Chain	−0.48	0.014
C8G	Complement C8 Gamma Chain	−0.48	0.014
AZGP1	Alpha-2-Glycoprotein 1, Zinc-Binding	−0.45	0.022
B2M	Beta2-microglobulin	−0.44	0.024
ITIH3	Inter-alpha-trypsin inhibitor heavy chain 3	−0.44	0.025
EFEMP1	fibulin-like extracellular matrix protein 1	−0.42	0.036
ITIH4	Inter-alpha-trypsin inhibitor heavy chain 4	−0.40	0.043
		Correlation (post-HD)	
IGLV8-61	Immunoglobulin Lambda Variable 8–61	0.42	0.036
ATRN	Attractin	0.60	0.001
PROC	Protein C	0.57	0.002
IGLV1-40	Immunoglobulin Lambda Variable 1–40	0.51	0.008
IGLV2-8	Immunoglobulin Lambda Variable 2–8	0.51	0.009
LCAT	Lecithin-cholesterol acyltransferase	0.49	0.012
C6	Complement component 6	0.49	0.012
FCN2	Ficolin	0.49	0.013
APOE	Apolipoprotein E	0.48	0.014
APOC3	Apolipoprotein C3	0.46	0.026
HGFAC	Hepatocyte growth factor activator	0.42	0.032
FETUB	Fetuin B	0.42	0.034
SERPINA10	Serpin Family A Member 10	0.42	0.035
CP	Ceruloplasmin	−0.40	0.047
C9	Complement component 9	−0.55	0.004
CD14	cluster of differentiation 14	−0.48	0.014
CFD	Complement Factor D	−0.44	0.026
C8B	Complement C8 Beta Chain	−0.41	0.037
PPBP	Pro-platelet basic protein	−0.41	0.038

**Table 5:** The biological pathways of the proteins exhibit correlation with 7αC4 in each of the before- and after- AD and HD conditions.

	Biological Process	Bonferroni *p*-Value	Proteins
Pre-AD			
Positive correlation	complement activation, classical pathway	0.0015	C3, IGHV6-1, CFI, IGHV3-15, CLU
	cholesterol metabolic process	0.0181	APOC1, APOE, APOB, APOL1
	lipoprotein metabolic process	0.0483	APOC1, APOE, APOL1
Pre-AD			
Negative correlation	no significant biological process uncovered		
Post-AD			
Positive correlation	adaptive immune response	<0.0001	IGHG1, IGKV1-16, IGHV3-23, IGKV1-12, IGHV3-15, IGKV1D-16, IGKV3-20
	immune response	<0.0001	IGKV1-16, IGHV3-23, IGKV1-12, IGKV1D-16, IGKV3-20
	immunoglobulin production	0.0136	IGKV1-16, IGKV1-12, IGKV3-20
	positive regulation of B cell activation	0.0168	IGHG1, IGHV3-23, IGHV3-15
	phagocytosis, recognition	0.0172	IGHG1, IGHV3-23, IGHV3-15
	phagocytosis, engulfment	0.023	IGHG1, IGHV3-23, IGHV3-15
	complement activation, classical pathway	0.0267	IGHG1, IGHV3-23, IGHV3-15
	B cell receptor signaling pathway	0.0315	IGHG1, IGHV3-23, IGHV3-15
Post-AD			
Negative correlation	negative regulation of peptidase activity	0.014	ITIH4, ITIH3, AMBP
Pre-HD			
Positive correlation	No discernible significant biological process revealed		
Pre-HD			
Negative correlation	positive regulation of immune response	0.0179	C8G, C8B, B2M
Post-HD			
Positive correlation	high-density lipoprotein particle remodeling	0.0074	APOC3, LCAT, APOE
	reverse cholesterol transport	0.0094	APOC3, LCAT, APOE
Post-HD			
Negative correlation	complement activation, alternative pathway	<0.0001	CFD, C9, C8B
	complement activation	0.0013	CFD, C9, C8B
	killing of cells of other organism	0.0172	C9, PPBP, C8B

## Data Availability

All mass spectrometry data are publicly available on ProteomeXchange repository (https://www.proteomexchange.org) (accessed on 12 December 2022) with the identifier PXD035386.

## Supplementary Materials

The following supporting information can be downloaded at: https://www.mdpi.com/article/10.3390/nu15224707/s1, Table S1: Identified 231 proteins in all the conditions (pre-AD, post-AD, pre-HD, and post-HD).
